# Prevalence of body dysmorphic disorder in the aesthetic medicine practice: a systematic review

**DOI:** 10.1192/j.eurpsy.2025.1856

**Published:** 2025-08-26

**Authors:** I. M. M. Peso Navarro, C. M. Cossio, C. G. Cerdan, M. L. Argudo, N. M. C. Espada, A. G. Mota, R. G. Juanes

**Affiliations:** 1 Psychiatry, Hospital Clínico Universitario de Salamanca, Salamanca; 2 Psychiatry, Hospital Clínico San Carlos, Madrid; 3 Psychiatry, Hospital Universitario de Son Espases, Mallorca, Spain

## Abstract

**Introduction:**

Body dysmorphic disorder (BDD) consists of preoccupation with one or more perceived defects or flaws in physical appearance that are not observable or appear slight to others, and which cause a great social deterioration. Its prevalence in aesthetic medicine is around 10 percent, and it is higher in women.

**Objectives:**

The overall objective of this systematic review is to gather integrated evidence to ascertain the prevalence of body dysmorphic disorder in an aesthetic medicine practice and its association with satisfaction with the results of the derived interventions.

**Methods:**

An initial search of ScienceDirect, PubMed, PsycInfo, Cochrane, and CINAHL was conducted. In addition, scientific journal publications, book articles and clinical guidelines were used. The search terms were: “body image”, “eating disorder”, and “aesthetic medicine”. The search included results from January 2000 to January 2023, in Spanish and English.

**Results:**

The total number of participants in all studies was 3004. 42.86% of the studies were conducted between 2000 and 2009, while the rest were conducted from 2010 onwards. It has been observed that most patients with BDD seek non-psychiatric treatment for their perceived appearance defects. The result after an aesthetic intervention is that in most patients with BDD there is no improvement in the concern for the perceived body image defect, and they present greater dissatisfaction. A high number of patients with BDD are being overlooked in aesthetic medicine practice who should receive a combined medical and psychological intervention.

**Conclusions:**

It is necessary to create protocols for an early diagnosis of body dysmorphic disorder in the aesthetic medicine practice which include a comprehensive approach.

**Disclosure of Interest:**

None Declared

